# Regulation of mitochondrial dynamics in 2-methoxyestradiol-mediated osteosarcoma cell death

**DOI:** 10.1038/s41598-020-80816-x

**Published:** 2021-01-15

**Authors:** Magdalena Gorska-Ponikowska, Paulina Bastian, Agata Zauszkiewicz-Pawlak, Agata Ploska, Adrian Zubrzycki, Alicja Kuban-Jankowska, Stephan Nussberger, Leszek Kalinowski, Zbigniew Kmiec

**Affiliations:** 1 Department of Medical Chemistry, Medical University of Gdansk, Debinki 1, 80-211 Gdansk, Poland; 2 Department of Biophysics, Institute of Biomaterials and Biomolecular Systems, University of Stuttgart, Stuttgart, Germany; 3 Euro-Mediterranean Institute of Science and Technology, Palermo, Italy; 4 Department of Histology, Medical University of Gdansk, Gdansk, Poland; 5 Department of Medical Laboratory Diagnostics, Medical University of Gdansk, Gdansk, Poland; 6 Biobanking and Biomolecular Resources Research Infrastructure Poland (BBMRI.PL), Gdansk, Poland

**Keywords:** Biochemistry, Cancer, Cell biology

## Abstract

Osteosarcoma (OS) is one of the most malignant tumors of childhood and adolescence. Research on mitochondrial dynamics (fusion/fission) and biogenesis has received much attention in last few years, as they are crucial for death of cancer cells. Specifically, it was shown that increased expression of the cytoplasmic dynamin-related protein 1 (Drp1) triggers mitochondrial fission (division), which activates BAX and downstream intrinsic apoptosis, effectively inhibiting OS growth. In the presented study, human OS cells (metastatic 143B OS cell line) were incubated with 2-methoxyestradiol (2-ME) at both physiologically and pharmacologically relevant concentrations. Cell viability was determined by the MTT assay. Confocal microscopy and western blot methods were applied to examine changes in Drp1 and BAX protein levels. Mitochondrial Division Inhibitor 1, MDIVI-1, was used in the study to further examine the role of Drp1 in 2-ME-mediated mechanism of action. To determine quantitative and qualitative changes in mitochondria, electron microscopy was used. 2-ME at all used concentrations increased mitochondrial fission and induced autophagy in OS cells. At the concentration of 1 µM 2-ME increased the area density of mitochondria in OS cells. Subsequent, upregulated expression of Drp1 and BAX proteins by 2-ME strongly suggests the activation of the intrinsic apoptosis pathway. We further observed 2-ME-mediated regulation of glycolytic state of OS cells. Therefore, we suggest that changes of mitochondrial dynamics may represent a novel mechanism of anticancer action of 2-ME. This finding may open new approaches to improve the efficacy of chemotherapy in the treatment of OS, however, it has to be confirmed by in vivo studies.

## Introduction

Osteosarcoma (OS) is one of the most malignant tumors of childhood and adolescence. Research on mitochondrial dynamics (fusion/fission) and mitochondrial biogenesis has received much attention in last few years, as these phenomena are crucial for understanding of many biological processes, including cancer cell death. Since accelerated mitochondrial fission (division) affects cell death in OS cells, many recent studies have explored relationships between mitochondrial dynamics, increased nitro-oxidative stress and apoptosis^1,2^. Specifically, it was shown that increased cytoplasmic dynamin-related protein 1 (Drp1) activated mitochondrial divisions (fission), leading to BAX recruitment and intrinsic apoptosis pathway activation what effectively inhibited OS growth^1^. Upon stimulation, Drp1 is activated and translocated into mitochondria, where it polymerizes leading to mitochondrial division^3^. In the cell, mitochondrial divisions exert various effects including cytochrome c release. One role of mitochondrial fission is to dispose damaged organelles by autophagy^4^. Notably, Drp1 mediates also activation of mitophagy, aspecific type of autophagy^5^. Growing evidence shows that mitophagy may take part in regulation of mitochondrial content, mitochondria divisions, metabolism as well as apoptotic signalling in cancer cells^6^. Importantly, mitochondrial stability defends the mitochondrial DNA maintenance as well as ATP generation^7^.

2-ME is a physiological metabolite of 17β-estradiol. 2-ME exerts a wide spectrum of anticancer activity proved by in vitro as well as in vivo studies^8–12^. 2-ME (branded as Panzem) has been under evaluation in different phases of clinical trials dedicated for several malignancies e.g. prostate cancer^13–16^. 2-ME serum concentrations are ranging between 30 pM in men to 30 nM in pregnant women^17–20^. As shown by clinical trials, recommended oral dose of Panzem is 1 g^15^. Thanks to established different forms of drug formulation e.g. nanoCrystal dispersion, the bioavaibility of the compound increased and Panzem could be used in clinical practice in future^15,16^. Pharmacological concentration of 2-ME stated as steady-state Cmax plasma concentration equals 21.7 µM. Therefore, pharmacological doses of 2-ME that are widely used in various experimental models range between nanomolar and micromolar concentrations. Notably, the minimum target dose of 2-ME, referred as low pharmacological relevant concentration, equals 11 nM^14,15^.

Previously, we showed that 2-ME selectively induces neuronal nitric oxide synthase (nNOS) and local generation of nitric oxide and its derivatives in nuclei of OS cells^9,11,21^. We further proved that 2-ME at both physiologically and pharmacologically relevant concentrations inhibited OS cell proliferation and migration due to regulation of mitochondrial biogenesis^22^. We also observed the induction of autophagy by 2-ME in OS cells^23^.

The aim of our present study was to further investigate the effects of 2-ME on mitochondrial dynamics in an experimental model of metastatic OS cell death.

## Materials and methods

### Reagents

Tissue culture media, antibiotic cocktail, fetal bovine serum, 2-ME, MDIVI-1 were purchased form Sigma Aldrich (Poland). Anti-mouse IgG were purchased from Abcam (UK). Mouse antibodies against Drp1 and BAX were produced by Santa Cruz Biotechnology (USA).

### Cell culture

OS 143B cells were cultured at 37˚C in a humidified atmosphere, saturated with 5% CO_2_. Minimum Essential Medium Eagle (MEM) was supplemented with 2 mM glutamine, 1% nonessential amino acids, and 10% heat-inactivated fetal bovine serum (FBS)^23^. In order to avoid the effects of glucose and Warburg effect in OS cells, low glucose, pyruvate and lactate-free MEM was chosen for cell culture and treatment^23^.

### Cell treatment

Treatments were performed in MEM without FBS to assess the potential physiological and pharmacological effect of 2-ME in OS 143B cells. OS cells were treated with 2-ME at physiologically (10 nM) and low pharmacologically (100 nM, 1 µM) relevant concentrations according to the experimental design. Control cells were treated with the solvent.

In the current study, we aimed to dissect the effect of 2-ME on the mitochondrial dynamics and mitochondria function at a time preceding OS cell death which correlates with local nitric oxide generation. Therefore, we chose 8 h incubation time strictly corresponding with the maximal peak of 2-ME-mediated generation of nitro-oxidative stress^11^.

### Cell viability assay (MTT)

The MTT assay was performed as previously described^9^. The results were presented as a percentage of control. Each experiment was performed at least three times.

### Electron microscopy

The OS 143B cells were seeded onto Petri dish at density of 1 × 10^6^ cells/plate. After 24 h of culturing in the standard medium, the cells were treated with 10 nM, 100 nM or 1 µM 2-ME for 8 h. Consequently, the cells were fixed in 2.5% glutaraldehyde in 0.1 mM sodium-cacodylate buffer, scratched and centrifuged. The cell pellets were then postfixated in 2% osmium tetroxide, dehydratated in ethanol and infiltrated with a mixture of propylene dioxide:epon/pure epon. The pelleted cells were subsequently embedded to polymerize. Finally, the ultra-thin sections (Reichert OmU3 ultramicrotome, Austria) were contrasted using uranyl acetate and lead citrate, and examined using transmission electron microscope at 100 kV (JEM 1200EX II, Japan).

### Morphometric analysis

The cytoplasm of randomly selected cells in each experimental group was outlined and its area measured in µm^2^ by using the ImageJ software (NIH, Bethesda, MD, USA), and the number of mitochondria in that area was counted. The results are shown as the number of mitochondria per unit area of the cytoplasm expressed in square micrometers.

### Fluorescence microscopy

The procedure was conducted by Image-iT Fix-Perm Kit (Life Technologies Corporation, USA). The cells were seeded at density of 3 × 10^5^ cells/well on chamber slides one day prior to the experiment. Then cells were treated with 10 nM, 100 nM or 1 µM 2-ME. After 8 h incubation, cell medium was removed and slides were washed 2 times with Wash Buffer with gentle agitation. The samples were incubated for 15 min in Fixative Solution. After removal of Fixative Solution, samples were washed 3 times with Wash Buffer. Next, Permeabilization Solution was added and the samples were incubated for 15 min and washed with Wash Buffer. After blocking for 60 min in Blocking Solution, the samples were incubated with anti-Drp1 primary antibody or anti-BAX primary antibodies (Santa Cruz Biotechnology, USA) diluted 1:200 in PBS with 0,1% BSA overnight at 4 °C with gentle agitation. Next day, the slides were washed in Wash Buffer with gentle agitation—3 times for 5 min. Then, they were incubated with secondary antibodies Goat Anti-Mouse IgG H&L (FITC) (Abcam, UK) diluted 1:500 in PBS for 60 min. After washing, the samples were counterstained with DAPI (1 µg/ml) for 10 min. Next the slides were closed with mounting medium and coverslips. On the next day digital cell images were captured with a confocal microscope (Opera Phenix, Perkin-Elmer, USA). The images were analyzed and merged using the ImageJ software (v1.52a, NIH, USA). From each well 10 representative images were chosen and 10 randomly chosen cells from each image were taken under further analysis given together a 100 of cells/sample. The fluorescence signal was measured as a mean value of fluorescence intensity from the whole area of each cell. The final fluorescence intensity of each sample is a mean value from a hundred of cells analyzed per each well. The fluorescence intensity was presented in relative fluorescence units (RFUs). Each experiment was performed at least three times.

### Determination of cellular ATP level

Determination of ATP level in samples was performed by Colorimetric ATP Assay Kit (ab83355, Abcam, UK). All the reagents such as ATP standard dilution, ATP Reaction Mix and Background Reaction Mix were prepared due to manufacturer’s protocol. Firstly, ATP standards of 50 µL (labelled from 1 to 6) were prepared to obtain standard curve. 1 × 10^6^ were seeded into 6-well plates and lysed with lyolysis on ice. Next, the samples were centrifuged for 5 min. at 4 °C at 13,000×*g* to remove any insoluble material. The supernatants were collected and transferred to new tubes. Before loading on 96-well plate, volume of all samples was adjusted to 50 µL with ATP Assay Buffer. 50 µL of Reaction Mix was added into each standard and sample wells and 50 µL of Background Reaction Mix was added into the background control sample wells. The plate was incubated at room temperature for 30 min protected from light. The absorbance was measured on a microplate reader (Tecan Group Ltd., Switzerland) at wavelength λ = 570 nm. The procedure was repeated at least 3 times.

### Western blot analysis

The analysis was performed as previously described^9^. Briefly, osteosarcoma 143B cells were seeded at a density of 1 × 10^6^ cells/10mm^2^ dish, cultured in standard medium for 24 h. After incubation according to the experimental design, cells were lysed. Total protein (20 μg/sample) was resolved by polyacrylamide gel electrophoresis using Amersham ECL 4–12% gels and transferred onto PVDF membrane. The membranes were then incubated with primary antibodies specific to Drp1 (1:2000) overnight at 4˚C, or β-Actin (1:50,000) for 30 min at room temperature. Chemiluminescence was detected using ImageQuant LAS 500 (GE Healthcare, Poland). The protein level was quantified by densitometry using Quantity One 4.5.2 software (Bio-Rad, Poland). The protein levels of Drp1, as determined by chemiluminescent signal quantification, were normalized to loading control, β-Actin. Each experiment was performed at least three times.

### Statistical analysis

The results are presented as the mean ± SD from at least three independent experiments. All microscopic evaluations were done on randomized and coded slides. Differences between control samples versus 2-ME-treated samples were assessed with one-way analysis of variance (ANOVA) with post hoc testing using a Dunnett’s multiple comparison test. A *p* value of less than 0.01 was considered to correspond with statistical significance. Data were analyzed using GraphPad Prism (GraphPad Software, Inc., version 6, USA).

## Results

### 2-ME induces morphological and quantitative changes in OS 143B cells

**Figure 1:**
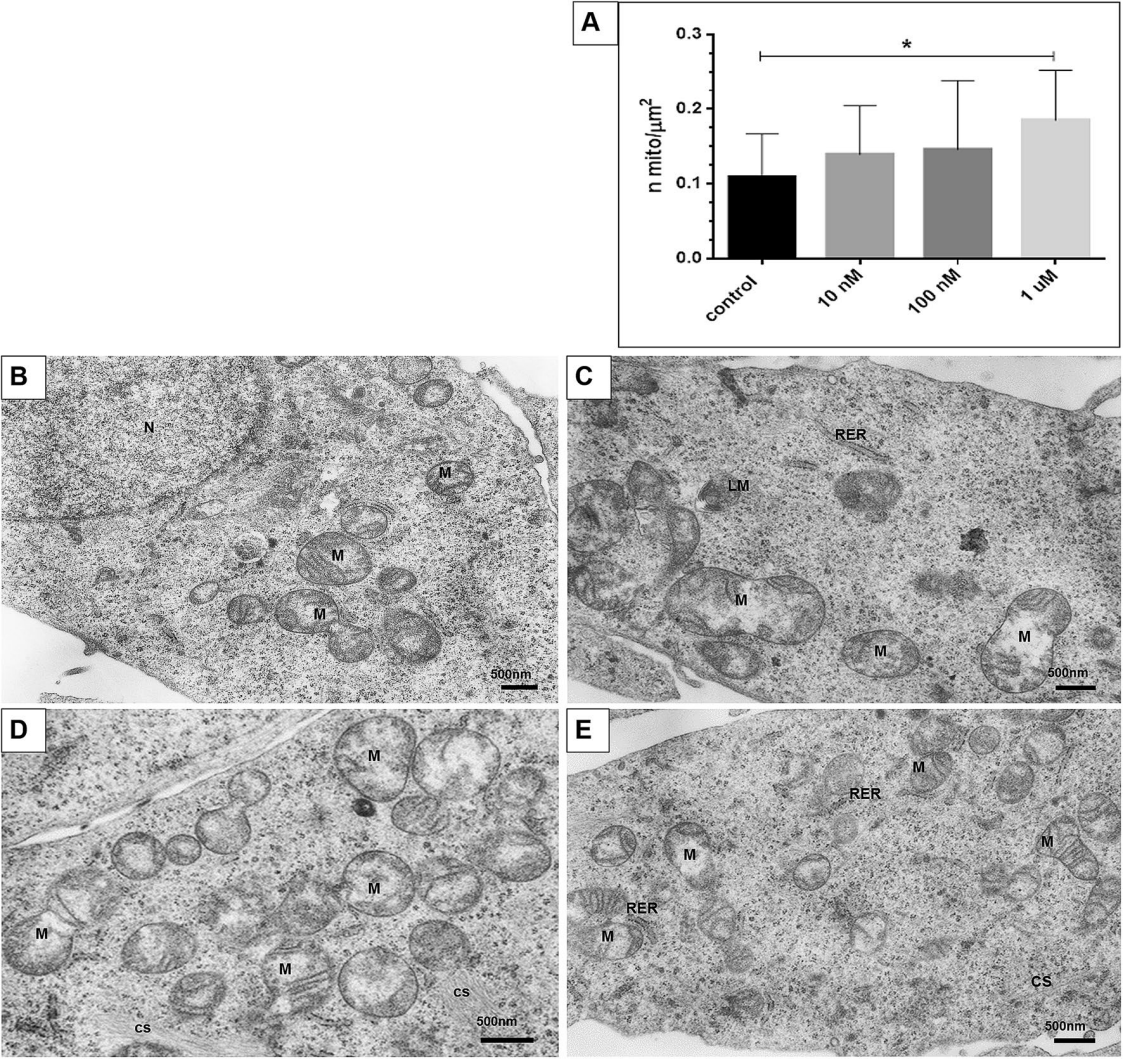
Electron microscopic analysis of osteosarcoma 143B cells treated with 2-ME. The cells were incubated in serum- and amino acid-free medium with 2-ME at the concentration of 10 nM, 100 nM and 1 µM 2-ME for 8 h, fixed and processed for transmission electron microscopy. (**A**) Morphometric analysis of mitochondria area density. 2-ME at the concentration of 1 µM significantly increased the number of mitochondria/µm^2^ of cytoplasm. Statistical analyses were performed using GraphPad Prism (v. 6.0; San Diego, CA, USA). After checking for the outlier values with the Grubbs’s test, the normal distribution of data sets was established with the Shapiro–Wilk test and Student’s t-test was used to assess statistical significance of the differences that was set at p < 0.05. Data present mean ± SD. * *p* < 0.05 vs control value. (**B**) Control cells contain moderate number of mitochondria (M), cell nucleus (N) with abundant euchromatin. (**C**) 2-ME, 1 µM. Abundant large mitochondria, lamellar body formation (LM), cisterna of rough endoplasmic reticulum (RER). (**D**) 2-ME, 100 nM. Numerous mitochondria in the cytoplasm, cytoskeletal filaments (cs). (**E**) 2-ME, 10 nM. Abundant mitochondria with thin, elongated cristae and electron-light matrix accompanied by RER. Magnifications: (**A**)—20,000×, (B–D)—10,000× .

**Figure 2:**
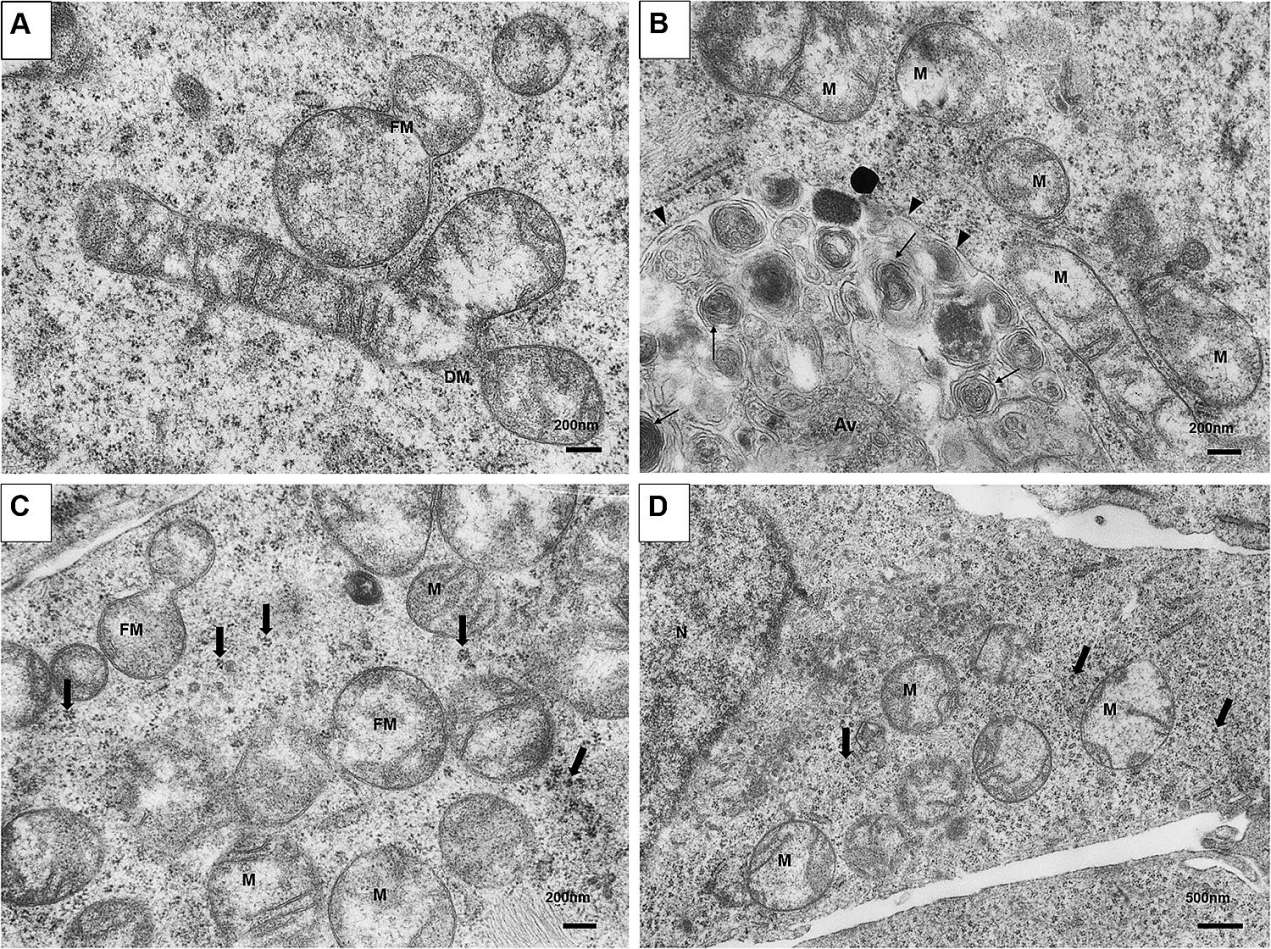
2-ME induces mitochondrial fission and authophagy in osteosarcoma 143B cells. The cells were incubated in serum- and amino acid-free medium with 10 nM, 100 nM and 1 µM 2-ME for 8 h, fixed and processed for transmission electron microscopy. (**A**) 2-ME, 1 µM. Mitochondrial fussion (FM) and division of mitochondria (DM). (**B**) 2-ME, 1 µM. Very large autophagic vacuole (Av), surrounded by double membrane (arrowheads) contains numerous lamellar bodies (thin arrows); presence of many mitochondria (M). (**C**) 2-ME, 100 nM. Numerous mitochondria, mitochondrial fission (FM), abundant glycogen rosettes (thick arrows). (**D**) 2-ME, 10 nM. Abundant mitochondria and glycogen rosettes, N—cell nucleus. Magnifications: (**A**–**C**)—20,000×, (**D**)—10,000×.

Electron microscopy (EM) demonstrated that mitochondria of OS 143B cells showed noticeable morphological and quantitative changes induced by the treatment with 2-ME (Fig. 1). The concentrations of 2-ME used throughout the manuscript are based on literature data^15–18^ and our previous studies^11,21^. We use 10 nM as the representative physiological dose, 100 nM as the representative low pharmacological dose and 1 µm as the representative pharmacological dose. To investigate whether 2-ME affected the number of mitochondria in the cultured OS cells we preformed morphometry (Fig. 1A). Since we did not make serial sections of the samples necessary to determine the absolute number of mitochondria in a given cell by classical stereology method [61], we measured the area density of mitochondria in all electron micrographs at the basal magnification of 5,000× . Morphometric analysis showed significantly increased number of mitochondria observed after 8 h treatment with 10 nM, 100 nM and 1 µM 2-ME (Fig. 1A). Treatment with both 10 nM and 100 nM 2-ME resulted in appearance of mitochondria with thin, elongated cristae and electron-light matrix accompanied by rough endoplasmic reticulum cisternae (Fig. 1B, D, E). We further observed mitochondrial fussion and division of mitochondria observed after incubation with 1 µM 2-ME (Fig. 2A). Moreover, 1 µM 2-ME not only increased the number of mitochondria, but also lamellar body formation was observed (Fig. 1C, and Suppl. Figure 1B, D). Smaller autophagic vacuoles (Suppl. Figure 1A) and a large autophagic vacuole surrounded with double membrane containing numerous lamellar bodies were observed after incubation with 1 µM 2-ME (Fig. 2B). Neither lamellar bodies nor increased number of mitochondria were detected in control cells (Fig. 1B). A closer inspection of EM-images revealed that the increased number of mitochondria after treatment with 2-ME may be caused by increased rate of mitochondrial divisions (fissions) (Fig. 2C). After treatment with 2-ME at all concentrations used, we observed presence of numerous glycogen rosettes (Fig. 2C, D). In cells treated with 1 µM 2-ME mitochondria were often associated with elongated cisternae of rough endoplasmic reticulum (Suppl. Figure 1C).

### 2-ME regulates the Drp1 protein expression in OS 143B cells

**Figure 3:**
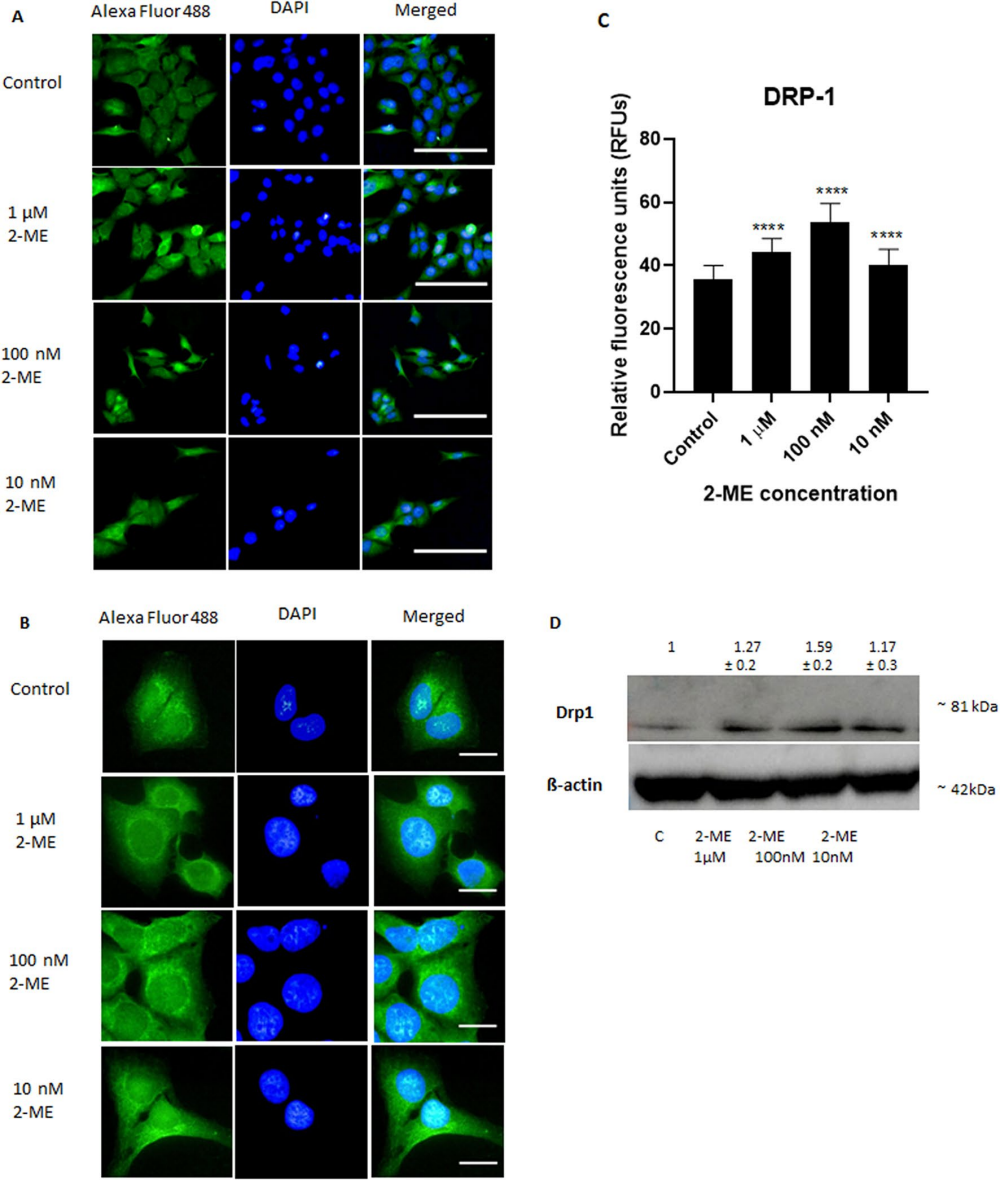
2-ME increases Drp1 protein expression in OS 143B cells. Representative immunofluorescence confocal images of OS 143B cells stained with Alexa Fluor 488-labeled anti-Drp1 antibody (green) and co-stained with nuclear stain (DAPI, blue). OS 143B cells were treated with 10 nM, 100 nM and 1 µM 2-ME for 8 h. Magnifications: (**A**) 200× (**B**) 1300 × Scale bars: A. 100 µm, B. 20 µm. (**C**) The fluorescence intensity of Drp1 after treatment with 2-ME. The images were analyzed and merged using the ImageJ software. The fluorescence intensity was presented in relative fluorescence units (RFUs). (**D**) Treatment with 2-ME at 10 nM, 100 nM, and 1 μM concentrations upregulates Drp1 protein level in OS 143B cells evaluated by Western blotting. Densitometric analysis of ratio Drp1/β-actin was performed using Quantity One 4.5.2 software. The presented immunoblots representative from one membrane are shown. Values are the mean ± SD of three independent experiments. ****p < 0.0001 versus control cells.

To examine whether the level of Drp1 in OS 143B cells is changed upon 8 h treatment with 2-ME, its expression was determined using the immunofluorescence technique. As shown in Fig. 3, the intensity of the Drp1 signal significantly increases dose-dependently in cells treated with 2-ME for 8 h (Fig. 3). Specifically, at the 2-ME concentrations of 10 nM, 100 nM, and 1 μM we observed significant increase of the fluorescence intensity of Drp1 by 12%, 40%, 23% compared with control cells, respectively (Fig. 3A–C). The results of western blot proved upregulation of Drp-1 induced by 2-ME. We observed as much as increase by 27%, 59%, 17% after treatment with 2-ME at 10 nM, 100 nM, and 1 μM concentrations (Fig. 3D).

**Figure 4:**
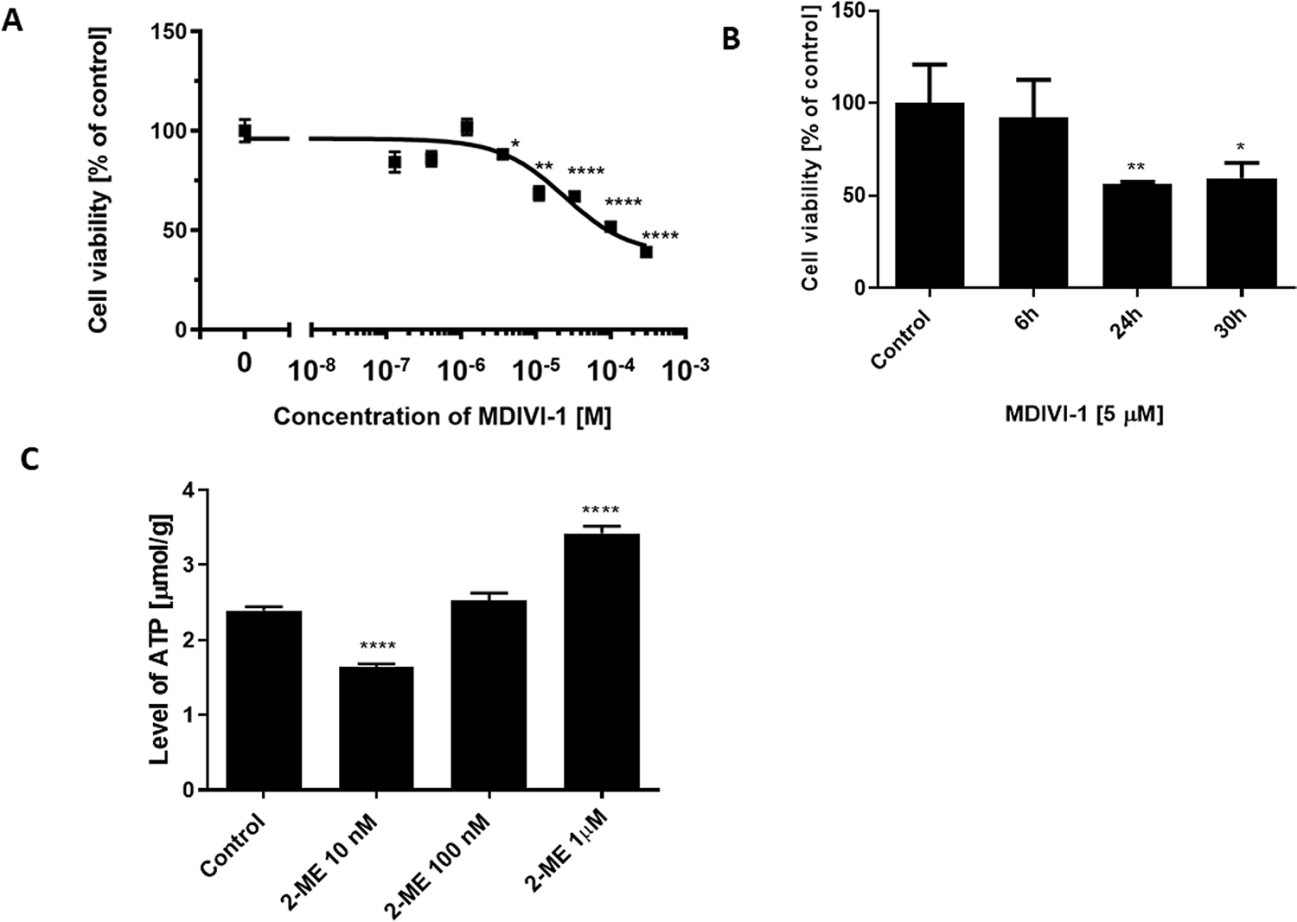
MDIVI-1 exerts cytotoxic effect in OS 143B cells. 2-ME regulates the ATP intracellular level in OS 143B cells. (**A**) MDIVI –1 significantly decreases OS 143B cell viability in a dose-dependent manner at concentrations higher than 10^–6^. OS 143B cells were treated with serial dilutions of MDIVI-1 within concentrations range of 300–0.1 µM for 24 h. The cell viability was determined by MTT assay. (**B**) Incubation of OS 143B cells with 5 µM MDIVI-1 1 for 6 h does not affect cell viability. (**C**) Changes in cellular level of ATP after 8 h-treatment of OS 143B cells with 10 nM, 100 nM, and 1 µM 2-ME. Values are the mean ± SD of three independent experiments. *p < 0.01, **p < 0.001, ***p < 0.0001, ****p < 0.00001 versus control cells.

Next, we consequently used a selective Drp1 inhibitor, MDIVI-1 in the study. We first evaluated the effect of treatment with MDIVI-1 on the proliferation of OS cells (Fig. 4A). OS 143B cells were treated for 24 h with serial dilutions of MDIVI-1 concentrations from 300 µM to 0.1 µM. As shown in Fig. 4A, MDIVI-1 exerts cytotoxic effects in OS 143B cells in a dose-dependent manner at concentrations higher than 10^–6^. For further studies, we chose 5 µM concentration of MDIVI-1 that did not significantly change the OS 143B cell viability after 6 h of incubation (Fig. 4B). In order to limit the cytotoxic effect of MDIVI-1, which at the 5 µM concentration was previously proved to significantly inhibit Drp1 guanosine triphosphatase (GTPase) activity in other experimental models^24–28^, we decided to pre-treat OS143B cells with this compound for 6 h.

**Figure 5:**
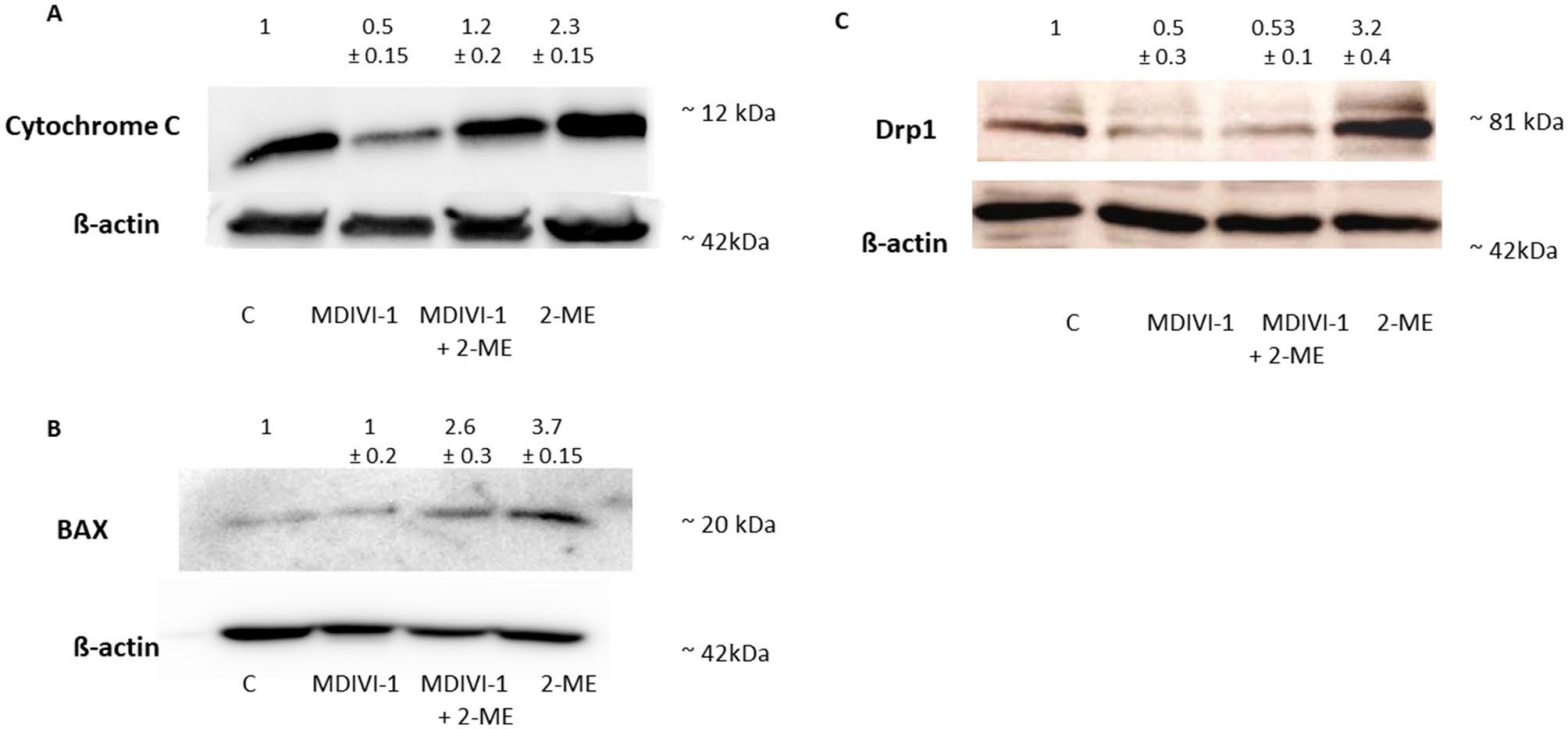
MDIVI-1 decreases the 2-ME-mediated upregulation of Drp1, BAX and cytochrome C. (**A**) Pre-treatment with 5 µM MDIVI-1 for 6 h decreases 2-ME-mediated upregulation of cytochrome C (**A**), BAX (**B**) and Drp-1 (**C**) proteins level in OS 143B cells evaluated by Western blotting. Densitometric analysis of ratio cytochrome C/β-actin, BAX/β-actin, Drp1/β-actin were performed using Quantity One 4.5.2 software. The presented immunoblots representative from one membrane are shown.

Consequently, we checked whether MDIVI-1 may influence the 2-ME-mediated effect on markers of mitochondrial apoptosis and Drp1 in OS 143B cells using Western blotting (Fig. 5A–C). For this part of the study, we used 2-ME at the concentration of 1 µM. As demonstrated, 2-ME upregulated both BAX and cytochrome C after 6 h of incubation by 370% and 230%, respectively (Fig. 5A, B). Treatment with 5 µM MDIVI-1 separately for 6 h does not significantly influence BAX, while slightly decreases cytochrome C expression (Fig. 5A, B). While, pretreatment with MDIVI-1 reversed stimulative effect of 2-ME on both BAX and cytochrome C (Fig. 5A, B).

As presented in Fig. 5C, treatment of the cells for 6 h only with 5 µM MDIVI-1 decreased Drp1 protein level by 50% compared to control. In consistency with the immunofluorescence results, 2-ME upregulated Drp1 protein level by 3.2-fold compared with control (Fig. 5C). However, this effect was almost completely abolished by the pretreatment of the cells for 6 h with 5 µM MDIVI-1 (Fig. 5C).

### 2-ME effects on ATP cellular concentration in OS 143B cells

Subsequently, we determined the effect of 8 h treatment of OS 143B cells with 2-ME on cellular ATP levels. We found that the effect of 2-ME on cellular ATP level was 2-phasic (Fig. 4C). 10 nM 2-ME decreased cellular ATP level in OS cells by 30%, however, 1 µM 2-ME increased the cellular level of ATP by 40% as compared to control cells (Fig. 4C).

### 2-ME increases BAX protein expression in OS 143B cells

**Figure 6:**
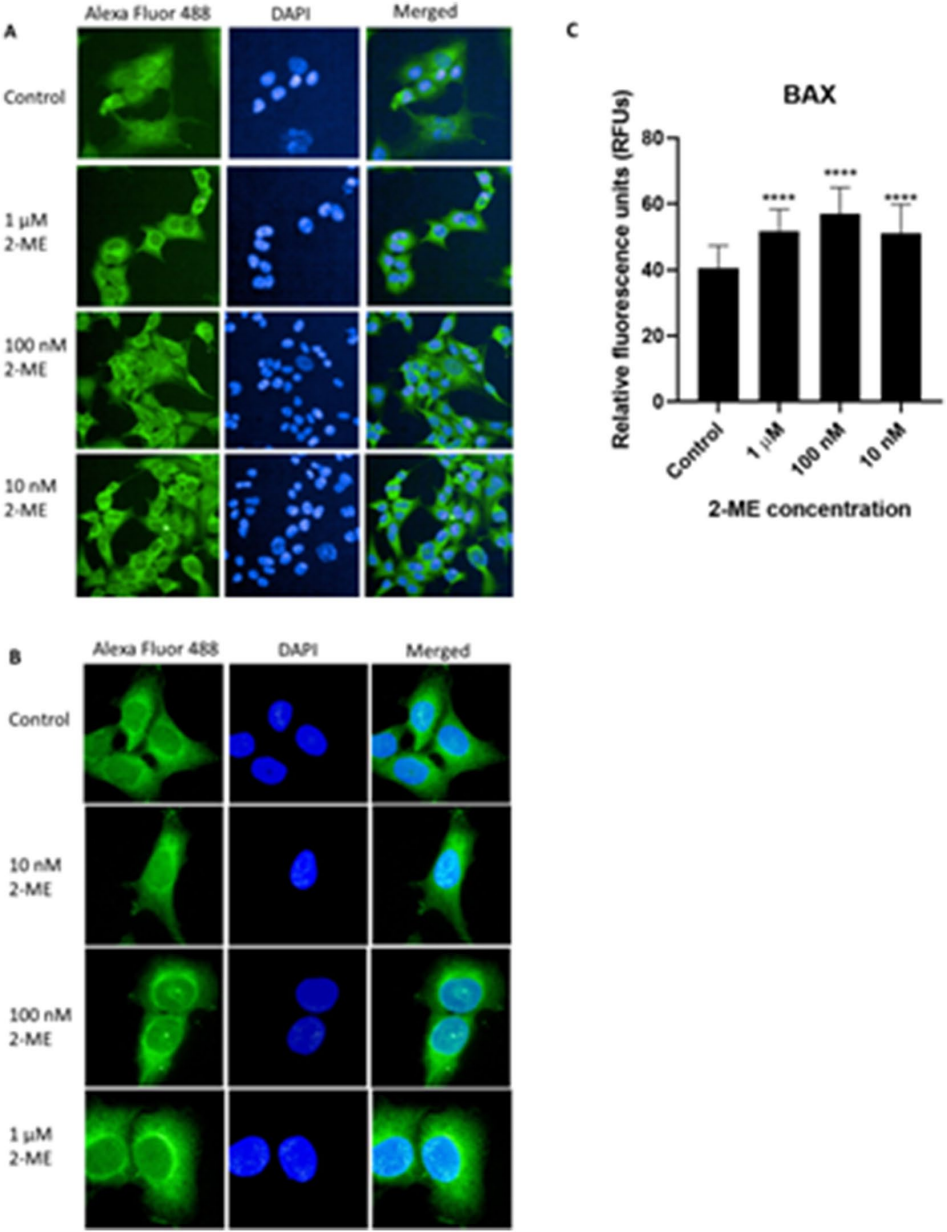
2-ME increases BAX protein expression in OS 143B cells. Representative immunofluorescence confocal images of OS 143B cells stained with Alexa Fluor 488-labeled anti-BAX antibody (green) and co-stained with nuclear stain (DAPI, blue). OS 143B cells were treated with 10 nM, 100 nM and 1 µM 2-ME for 8 h. Magnifications: (**A**) 200× , (**B**) 1300× Scale bars: (**A**) 100 µm, (**B**) 20 µm. (**C**) The fluorescence intensity of BAX protein after treatment with 2-ME. The images were analyzed and merged using the ImageJ software. The fluorescence intensity was presented in relative fluorescence units (RFUs). Values are the mean ± SD of three independent experiments. ****p < 0.0001 versus control cells.

In our previous studies we confirmed that 2-ME exerts pro-apoptotic effect in OS 143B cells within nM—µM concentrations range^11^. Herein, we investigated how 2-ME affects the expression of pro-apoptotic BAX protein in OS 143B cells as assessed by the immunofluorescence technique. We observed that 8 h treatment of cells with 10 nM, 100 nM, and 1 μM 2-ME resulted in an increase of BAX expression measured as a fluorescence intensity by 26%, 42%, and 27%, compared with control cells, respectively (Fig. 6A–C).

## Discussion

We have previously established that 2-ME exerts an anticancer effect at both physiologically (nM) and pharmacologically (µM) relevant concentrations by selective induction of nNOS, nuclear recruitment of this enzyme, and subsequent nitro-oxidative stress generation in the nuclei of OS cells^11,21^.

We showed that the consequence of 2-ME-mediated nuclear hijacking of nNOS is inhibition of mitochondrial biogenesis that was dependent on the used concentrations of 2-ME^22,29^. The aim of the current study was to further dissect the effects of 2-ME in OS cell death model, focusing on possible regulation of mitochondrial homeostasis. Under pathological conditions, including cancer, accumulation of dysfunctional mitochondria leads to oxidative stress and impairs cell function^30^. This phenomenon drew attention to two processes, mitochondrial dynamics and mitophagy, recognized as two crucial processes underlying mitochondrial homeostasis^31^. Morphological and bioenergetic characterization of the life cycle of an individual mitochondrion reveals several points where biogenesis, fusion, fission, and mitophagy interact^32^. As showed in this report, 2-ME-mediated changes in mitochondrial content of OS cells are manifested by an increased number of small mitochondria. Up to date, the role of 2-ME in the regulation of mitochondrial dynamics and mitochondrial content has not been investigated. Taking together the previously observed inhibition of mitochondrial biogenesis and decrease in mitochondrial mass in OS 143B cells after 24 h treatment with 2-ME^22^, the increased number of small mitochondria by 2-ME strongly suggest induction of mitochondrial fission^33^. Mitochondrial fission plays a variety of different functions including release of cytochrome c during apoptosis^34,35^. It also serves to eliminate damaged organelles from the constantly changing mitochondrial network in order to allow their removal by mitochondria selective autophagy, i.e. mitophagy. As suggested, mitochondrial fission facilitates the selective mitophagy of protein aggregates. Whether mitophagy leads to tumor promotion or tumor suppression seems to be highly dependent on the cancer type and microenvironmental context^6^. Previously, 2-ME-induced autophagy was reported as one of anticancer mechanisms in different cancer cell types, including OS 143B cell line^23^. In our study electron microscopy clearly showed that 2-ME increased the number of mito-phagosomes. Additionally, lamellar body formation, typical for lysosomes^36^, was observed after treatment with 2-ME what indicates autophagy. Indeed, increased mitophagic activity is strictly correlated with decrease in mitochondrial mass^37^. Noteworthy, decrease in mitochondrial mass was observed after treatment with 2-ME^22^. To efficiently induce mitophagy, beyond induction of the mitochondrial fission, mitochondria have to be dysfunctional and/or depolarized^38^. Therefore, our demonstration of mitophagy induction by 2-ME is supported by previously observed 2-ME-mediated decreased mitochondrial membrane potential in OS 143B cells^22^. We have also previously proved that OS 143B cells treated for 24 h with 2-ME are characterized by increases in monomeric tubulin background and a distinct decrease in the number of microtubules. 2-ME was thus stated to have colchicine-like-destabilizing effects on microtubules^29^. Replication of mitochondrial DNA (mtDNA) was also suppressed in 2- ME-treated cells^29^. Indeed, mitophagy also eliminates mitochondria during cytoplasmic remodeling and degrades mitochondrial DNA (mtDNA), including damaged and mutated mtDNA^39^.

Both mitochondrial fission and mitophagy machineries are mediated by Drp1 protein, a member of the dynamin family of GTPases^5,29,35,40^. Indeed, we showed that Drp1 constitutes a molecular target for 2-ME in the established model of OS cells mimicking cancer metastasis. During mitochondrial fission Drp1 is recruited from the cytoplasm to the mitochondria, where it interacts with its receptors in the outer mitochondrial membrane. It has been further reported that mitochondria need to be fragmented prior to engulfment of phagophores into autophagosomes and activation of Drp1-mediated mitochondrial autophagy^7^. It was further shown that increasing the Drp1 expression activates mitochondrial fission, which directly results in the translocation of BAX protein into mitochondria and downstream intrinsic apoptosis, effectively inhibiting growth of OS cells^1^. In consistency with our findings, it was previously shown that anticancer agent, cryptotanshinone, activated Drp1-mediated mitochondrial fission in OS cells resulting in mitochondrial fragmentation and induction of apoptosis^1^. Moreover, similarly to our results, cryptotanshinone induced Drp1 expression, which contributed to driving BAX translocation from the cytosol to the mitochondria, effectively inhibiting growth of OS cells^1,41^. We confirmed the observed effect of 2-ME on Drp1 protein by the pre-treatment of the cells with the selective inhibitor of Drp1 activity, being also an established inhibitor of mitochondrial fission, MDIVI-1^24^. However, it has to be noted that other authors suggested, that the inhibition of Drp1 may not be solely mechanism of MDIVI-1 activity, especially at the concentration of MDIVI-1 higher than 25 µM^24,42^. This fact seems to be associated with cytotoxic effect of MDIVI-1 on cancer cells, including lung (H460 and A549) and colon cancer (HCT116) cell lines^43^. Indeed, in our experimental model the cytotoxic effect of MDIVI-1 was also observed. Therefore, in order to limit the mode of action of MDIVI-1 only to Drp1 inhibition, we used in the study non-cytotoxic, 5 µM MDIVI-1 concentration, previously found to effectively inhibit mitochondrial fission^20–24^. Notably, we demonstrated that pre-incubation with MDIVI-1 significantly reduces 2-ME-activated BAX and cytochrome C. Indeed, completion of BAX recruitment and cytochrome C release strictly correlates with mitochondrial fission during apoptosis^44,45^. As we previously published, the percent of apoptosis in the presence of 2-ME ranging from concentrations of 10^−10^ M to 10^−6^ M after 24 h treatment is at similar level—about 10% of apoptotic 143B cells^11^. While, treatment of 143B OS cells with 10^−5^ M 2-ME results in a dramatic 40% increase in apoptotic cell number in comparison to the control cells^11^. Therefore, the obtained results confirms that mitochondria fission is one of pro-apoptotic mode of action of 2-ME. However, the precise role of mitochondrial fission during apoptosis remains elusive^46^ and further research needs to be performed.

Dose-dependent activity of 2-ME concentrations on the expression of DRP-1 and BAX may be explained by various effects of NO related to its concentration or time of exposure^47^. Such differences between activity of different doses of 2-ME has also been previously observed^22^. We proved that physiological relevant concentrations of 2-ME down-regulates PGC-1α, thus, decreasing mitochondrial mass, and lowering expression of COXI in osteosarcoma cells^22^. In contrast, 2-ME at higher doses only slightly reduces mitochondrial mass while does not impact on both PGC-1α and COXI^22^. Previously, we have presented that incubation of osteosarcoma cells with 10 nM and 100 nM concentrations of 2-ME for 8 h more potently increases nuclear NO levels as compared with higher doses of the compound^11,47^. Noteworthy, it was shown in Alzheimer’s disease (AD) model that NO induces S-nitrosylation of the Drp-1 resulting in excessive divisions of mitochondria and induction of cell death^48^. It was further evidenced in AD model that Drp-1 S-nitrosylation leads to its hyperactivation, fragmentation of mitochondria and bioenergetic compromise. So far, the impact of NO on Drp-1 in cancer cells remains to be elucidated. However, we can suspect these mechanisms are in common. The stronger activity of lower concentrations of 2-ME on mitochondria was previously observed^22^. We previously demonstrated that 2-ME inhibits mitochondrial biogenesis, especially at physiological concentrations, as a consequence of the nuclear recruitment of nNOS in osteosarcoma cell death model.

Notably, besides its impact on mitochondrial dynamics and cell death, Drp1 has also been associated with metabolic regulation^37^. Dynamic stability of mitochondria allows cells to preserve mitochondrial genome integrity and generate ATP^7^. It was reported that tumor tissues contain much higher amount of ATP compared to healthy tissues, in which ATP would be basically undetectable because it is produced on-demand, i.e. only when needed^49^. The observed here opposite activity of 10 nM and 1 µM 2-ME on the total pool of ATP cellular level may be explained by concentration dependent-different rate of cell death. Notably, extracellular ATP accumulates in the extracellular space of dying tumor cells, while the intracellular concentration of ATP declines before and/or during the cell death process^49^. Multiple studies report anticancer activity of ATP via its binding to purinergic receptors^50,51^. Indeed, the inhibitory effect of ATP on growth of various cancer cell types was shown in prostate and breast cancers as well as melanoma^51,52^. Of note, it was shown that intravenous ATP increases the survival rate in clinical trials in patients with pre-terminal cancer^53^. On the other side, activity of ATP via purinergic receptors may mediate pro-tumorigenic effects in prostate and breast cancer cells^51,53^. Importantly, in consistency with our study, it was previously reported that 2-ME at 0.5 µM concentration suppresses glycolytic state of melanoma 435R cells via increasing the cellular ATP level and inhibiting extracellular lactate content^54^. Indeed, we recently also showed that 2-ME abrogated pro-migratory and pro-proliferative potential of L-lactate in OS 143B cells^23^.

By means of electron microscopy, we also observed the numerous glycogen rosettes in 2-ME-treated OS cells. The contribution of glycogen metabolism to carcinogenesis, cancer cell growth, metastasis, and chemoresistance is poorly understood^55^. The levels of glycogen have been found to correlate with biological processes in metabolic reprogrammed cancer cells^56,57^. On the other side, glycogen has a crucial role to promote cancer cell survival under hypoxic conditions^54^ . Previously, we showed that 2-ME impacts on amino acids‐mediated metabolic reprogramming in OS cells leading to cell death^58^. Therefore, increasing glycogen rosettes by 2-ME may be considered as a mechanism of tumor adaptation to anticancer treatment. Nonetheless, the importance of 2-ME-mediated increase in glycogen rosettes needs to be further investigated.

## Conclusions and future directions

To reassume, in the current study we showed that 2-ME at both physiologically and pharmacologically relevant concentrations regulate the DRP1 and BAX protein levels as well as cellular ATP content in metastatic OS cells. Based on the current and previously obtained studies^11,22,23^, we suggest that anticancer mechanism of 2-ME relies on selective nitro-oxidative stress generation controlling the mitochondrial dynamics, including inhibition of biogenesis and induction of mitochondrial fission, finally resulting in mitophagy and cancer cell death. Taking into consideration that the crucial role of the purinergic receptor, P2X7R, in pathogenesis of OS was found^59^, it is thus tempting to speculate that the regulation of cellular ATP level might be used in designing effective anticancer OS chemotherapy.

## Supplementary Information

Supplementary Figure 1.

## Data Availability

The data used to support the findings of this study are available from the corresponding author upon request.
