# Supplementary material for: Regulation of mitochondrial dynamics in 2-methoxyestradiol-mediated osteosarcoma cell death
Source: Sci Rep. 2021 Jan 15;11:1616. doi: 10.1038/s41598-020-80816-x (PMC7811003; doi:10.1038/s41598-020-80816-x)
Supplement: Supplementary file 1 — Supplementary Figure 1. [file 41598_2020_80816_MOESM1_ESM.docx]

**Regulation of mitochondrial dynamics in 2-methoxyestradiol-mediated osteosarcoma cell death**

**Magdalena Gorska-Ponikowska#*^1,2,3^, Paulina Bastian#^1^, Agata Zauszkiewicz-Pawlak^4^, Agata Ploska^5,6^, Adrian Zubrzycki^4^, Alicja Kuban-Jankowska^1^, Stephan Nussberger ^2^, Leszek Kalinowski^5,6^, Zbigniew Kmiec^4^**

1. Department of Medical Chemistry, Medical University of Gdansk, Poland;
2. Department of Biophysics, Institute of Biomaterials and Biomolecular Systems, University of Stuttgart, Stuttgart, Germany;
3. Euro-Mediterranean Institute of Science and Technology, Palermo, Italy;

^4^ Department of Histology, Medical University of Gdansk, Poland;

^5^ Department of Medical Laboratory Diagnostics, Medical University of Gdansk, Gdańsk, Poland

^6^ Biobanking and Biomolecular Resources Research Infrastructure Poland (BBMRI.PL), Gdańsk, Poland

# Equally contributed to the study

^*^ Correspondence: Magdalena Gorska-Ponikowska, Department of Medical Chemistry, Medical University of Gdansk, Debinki 1, 80-211 Gdansk, Poland, tel: +48 583491450; [magdalena.gorska-ponikowska@gumed.edu.pl](mailto:magdalena.gorska-ponikowska@gumed.edu.pl)


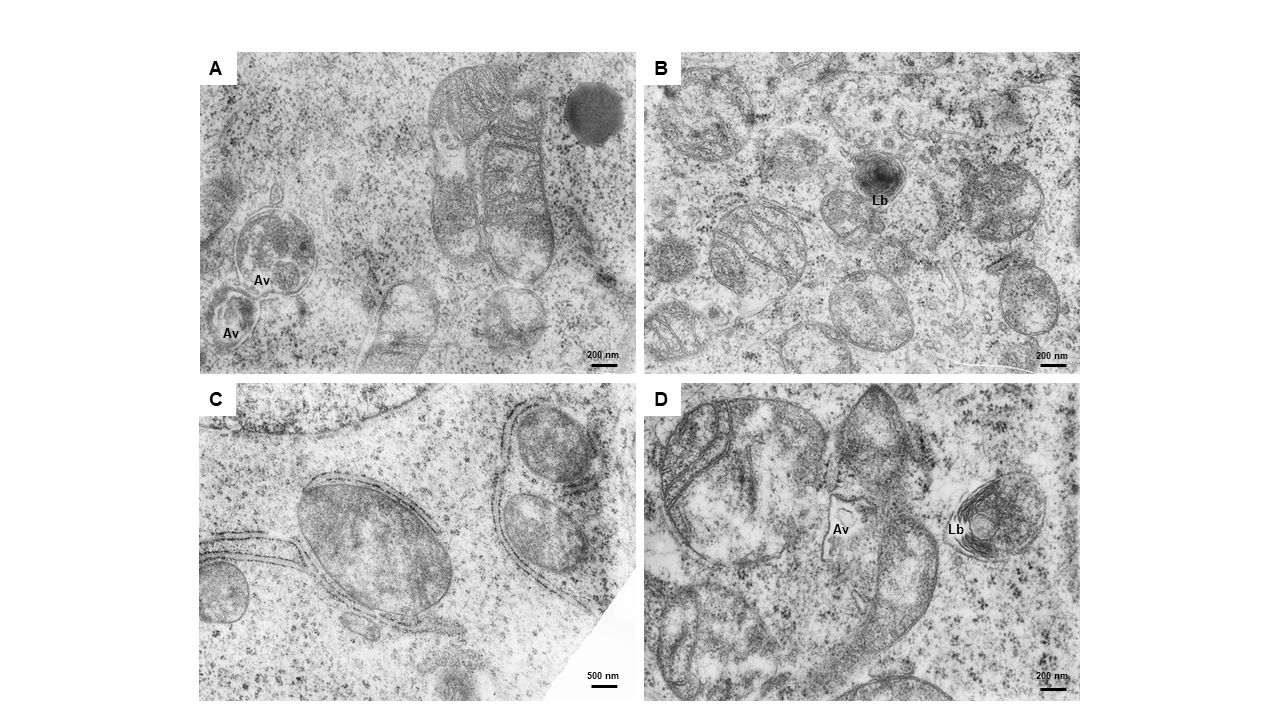


Fig.1 Electron microscopic analysis of osteosarcoma 143B cells treated with 2-ME. A. Control osteosarcoma 143B Cells. The cells were incubated in serum- and amino acid-free medium with 2-ME at the concentration of 10 nM (B), 100 nM (C) and 1 µM 2-ME (D) for 8 h, fixed and processed for transmission electron microscopy. Electron micrographs are obtained at magnifications (20-30K).


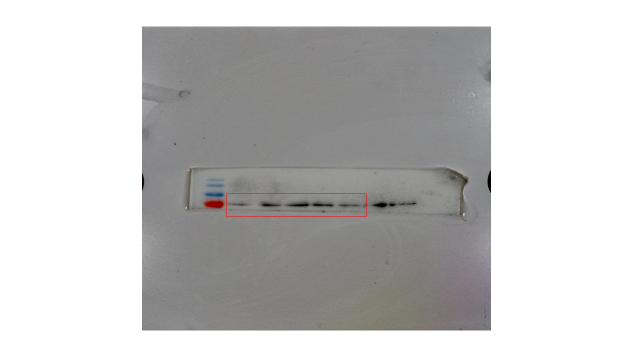


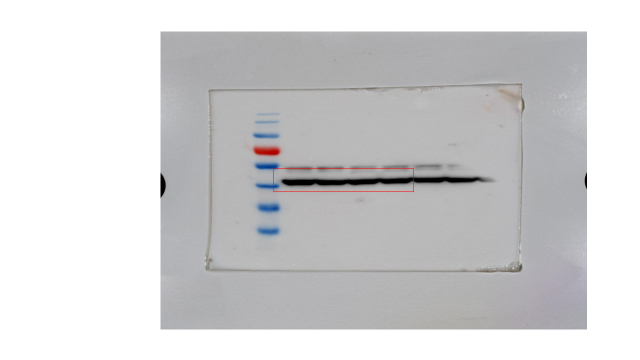


Fig. 2 Treatment with 2-ME at 10 nM, 100 nM, and 1 μM concentrations upregulates Drp1 protein level in osteosarcoma 143B cells evaluated by Western blotting. Drp1 and β-actin protein bands, respectively, are indicated in red. Original blots are presented.


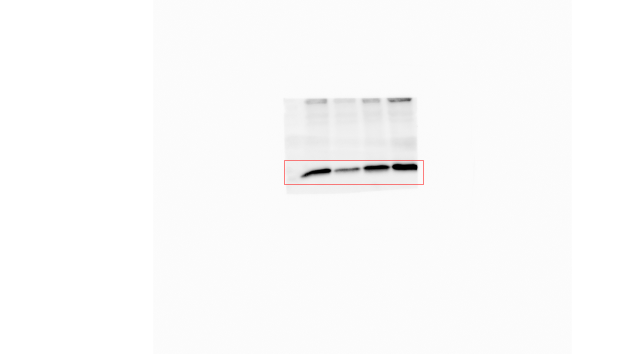

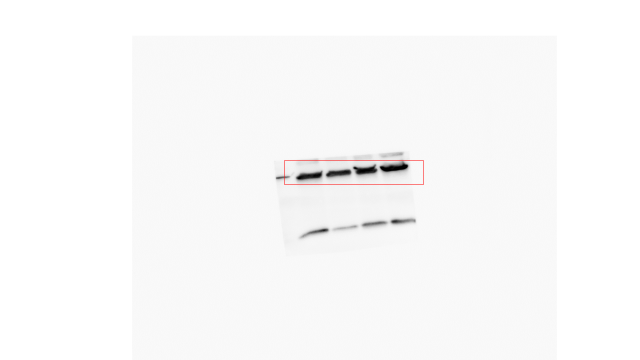


Fig. 3. Pre-treatment with 5 µM MDIVI-1 for 6 h decreases 2-ME-mediated upregulation of cytochrome C protein level in OS 143B cells evaluated by Western blotting. Cytochrome C and β-actin protein bands, respectively, are indicated in red. Original blots are presented.


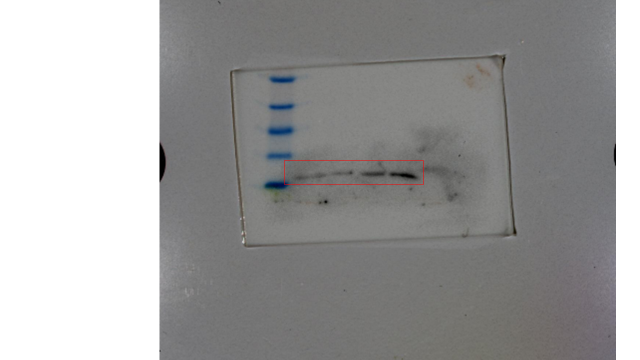


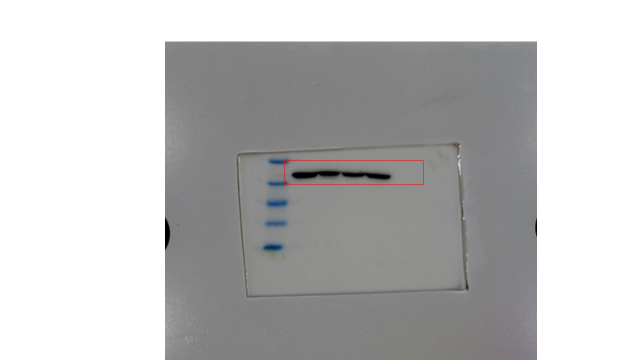


Fig. 4

Pre-treatment with 5 µM MDIVI-1 for 6 h decreases 2-ME-mediated upregulation of BAX protein level in OS 143B cells evaluated by Western blotting. BAX and β-actin protein bands, respectively, are indicated in red. Original blots are presented.


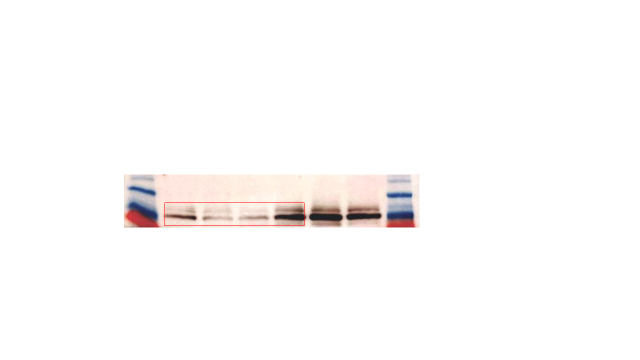


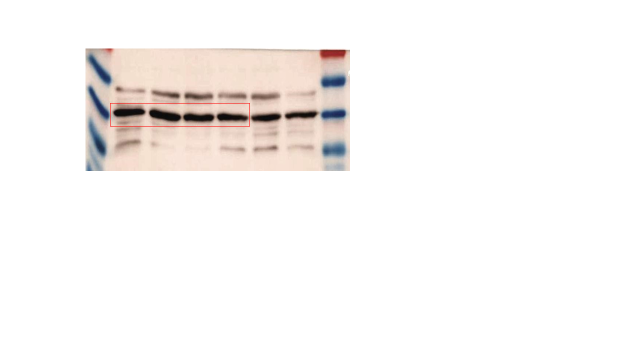
Fig.5

Pre-treatment with 5 µM MDIVI-1 for 6 h decreases 2-ME-mediated upregulation of Drp-1 protein level in OS 143B cells evaluated by Western blotting. Drp 1 and β-actin protein bands, respectively, are indicated in red. Original blots are presented.
